# Dihydromyricetin Mitigates Depression-Related Memory Impairments Through Regulation of Hippocampal PKA-CREB-BDNF Pathway in Mice

**DOI:** 10.3390/biology15141144

**Published:** 2026-07-14

**Authors:** Le Wang, Yuxiang Wang, Hao Chen, Zhiming He, Yanping Long, Lisha Yu, Chuli Xiao, Jiaxiu Zhou, Xudong Yu, Qingshan Long, Xinhua Shu

**Affiliations:** 1 The Brain and Behavior Laboratory, Pu Ai Medical School, Shaoyang University, Shaoyang 422000, China; wangle2021@hnsyu.edu.cn (L.W.); yuxiangwangsearch@163.com (Y.W.); chenhao_neuro@163.com (H.C.); 40003@hnsyu.edu.cn (Z.H.); 4317@hnsyu.edu.cn (Y.L.); 4315@hnsyu.edu.cn (L.Y.); xcl_neuro@163.com (C.X.); 3910@hnsyu.edu.cn (X.Y.); 2 The Brain Cognition and Brain Disease Branch, Pu Ai Medical School, Shaoyang University, Shaoyang 422000, China; 3 Department of Anesthesiology, Central Hospital of Shaoyang, Shaoyang 422000, China; zjx0413@126.com; 4 Department of Neurosurgery, Zhongshan Torch Development Zone People’s Hospital, Zhongshan 528400, China; 5 Department of Biological and Biomedical Sciences, Research Centre for Health, School of Health and Life Sciences, Glasgow Caledonian University, Glasgow G4 0BA, UK

**Keywords:** dihydromyricetin, chronic restraint stress, depression, memory impairment, PKA-CREB-BDNF pathway, network pharmacology

## Abstract

Background: Depression-associated memory impairment is a clinically significant comorbidity that responds poorly to conventional antidepressants. Dihydromyricetin (DHM), a natural dihydroflavonoid, exhibits neuroprotective and antidepressant-like effects, but its impact on stress-induced memory deficits and the underlying mechanisms remain unclear. Methods: A chronic restraint stress (CRS) mouse model was employed. DHM was intraperitoneally administered daily (20 mg/kg bodyweight) for 8 consecutive days. Depression-like behavior and memory functions were assessed with the tail suspension test, the object recognition memory (ORM) task, and the Y-maze test. Network pharmacology, molecular docking, and molecular dynamics (MD) simulations were integrated to predict potential targets and associated signal pathways. Expression of hippocampal PKA, p-CREB, CREB, and BDNF was measured through Western blotting. Results: CRS significantly increased immobility time in the tail suspension test and impaired both recognition memory (ORM) and spatial working memory (Y-maze), without affecting locomotor activity. DHM treatment effectively reversed behavioral despair and memory deficits. Network pharmacology identified 47 intersecting targets between DHM and depression-related memory impairment, with CREB1, ESR1 and NOS1 as core hub nodes. KEGG enrichment particularly implicated the involvement of the cAMP signaling pathway and other signaling pathways, which are associated with depression and/or memory impairment. Molecular docking predicted strong binding affinities of DHM to CREB1 (−9.225 kcal/mol), NOS1 (−9.113 kcal/mol) and ESR1 (−7.903 kcal/mol). MD simulations confirmed the thermodynamic stability of DHM-ESR1 and DHM-NOS1 complexes. Western blot analysis demonstrated that DHM treatment significantly increased the levels of hippocampal PKA, p-CREB and BDNF, without altering total CREB expression in CRS mice. Conclusions: DHM alleviated CRS-induced depression-like behaviors and memory deficits, possibly through regulation of multiple signaling pathways, including the hippocampal PKA-CREB-BDNF signaling pathway. These findings highlight DHM as a promising natural therapeutic candidate for depression-associated cognitive impairment.

## Simple Summary

Depression is a common mood disorder, affecting 280 million individuals globally. Memory impairment has emerged as a clinically significant comorbidity of depression and is attracting growing attention. Given that current antidepressant treatments, including inhibitors of selective serotonin reuptake and tricyclic antidepressants, are generally ineffective in treating depression-related cognitive dysfunction, new treatment is needed urgently. Dihydromyricetin (DHM) is a natural flavonoid, mainly distributed in vine tea. DHM has demonstrated capacity against a wide range of disorders, including diabetes, cardiovascular disease, liver disease, kidney injury, cancer and neurodegenerative diseases. However, there is limited study of DHM in depression-associated memory impairment. Here we report that DHM alleviated chronic restraint stress induced depression-associated memory impairment in mice. We also used network pharmacology to find that DHM has multiple targets and mediates multiple signaling pathways; we validated one of the predominant signaling pathways, the PKA-CREB-BDNF pathway. Our data sheds new light on the molecular mechanisms involved in depression-associated cognition deficit and suggests that DHM has therapeutic potential to treat patients with depression.

## 1. Introduction

Depression is a serious and widespread mental disorder and a leading cause of disability worldwide [1]. The global COVID-19 pandemic has substantially contributed to the increased prevalence of depression [2]. Beyond core symptoms such as depressed mood, anhedonia, feelings of despair, and excessive guilt, memory impairment has emerged as a clinically significant comorbidity attracting growing attention [3]. Current antidepressant treatments, including inhibitors of selective serotonin reuptake and tricyclic antidepressants, are generally ineffective in treating depression-related cognitive dysfunction [4]. Therefore, the identification of effective active compounds with fewer side effects for the treatment of depression-associated memory impairment remains an urgent clinical need.

Dihydromyricetin (DHM), also known as ampelopsin, is a natural dihydroflavonoid extracted from the vine tea (*Ampelopsis grossedentata*). Accumulated data has demonstrated that DHM exhibits diverse pharmacological characteristics, including antioxidant, anti-inflammatory, and anti-tumor effects [5]. Large amounts of evidence suggest that DHM exerts neuroprotective activity [6]. For instance, DHM enhanced cell viability and reduced cytotoxicity, oxidative stress, and apoptosis following oxygen glucose deprivation and reoxygenation in HT22 cells, indicating its neuroprotective potential [7]. DHM also attenuated apoptosis and astrogliosis, alleviating cerebral ischemia–reperfusion injury [8]. Moreover, DHM has been shown to relieve symptoms of comorbid diabetic neuropathic pain and depression [9,10] and exert antidepressant-like effects in lipopolysaccharide (LPS)-induced, chronic unpredictable mild-stress-induced, and chronic corticosterone-induced depressive models [11,12,13]. Importantly, DHM alleviated memory impairments in models of Huntington disease [14] and Alzheimer’s disease [15], as well as memory deficits induced by social isolation [16], acute sleep deprivation [17], and hypobaric hypoxia [18]. However, whether DHM improves stress-induced memory deficits, particularly depression-related memory impairment, has not been systematically investigated.

Chronic restraint stress (CRS) is a well-established stress paradigm that induces dysregulation of the hypothalamic–pituitary–adrenal (HPA) axis, increased plasma corticosteroid levels, and downregulated expression of hippocampal glucocorticoid receptor (GR), which plays a critical role in both depression and memory regulation [19]. CRS reliably induces memory impairments and depression-like behaviors in rodents, making it a widely used model for studying the comorbidity of depression and cognitive dysfunction [19]. In this study, we first investigated whether DHM attenuates comorbid depression and memory impairments in a CRS mouse model. Furthermore, we employed network pharmacology analysis to predict the potential mechanisms underlying DHM’s therapeutic effects. Among several predicted signaling pathways, we experimentally validated the involvement of the hippocampal PKA-CREB-BDNF pathway in DHM’s beneficial effects.

## 2. Materials and Methods

### 2.1. Animals

Historically, many studies have favored male mice due to concerns that female mice exhibit greater variability in depression-like behaviors caused by hormonal fluctuations during the estrous cycle. Researchers think that this cyclic variation may introduce experimental “noise,” reduce result consistency, and increase statistical complexity. Therefore, we also used male mice for this study. Male (Institute for Cancer Research (ICR) mice (age of 7 weeks, body weight: 28–32 g) were purchased from Hunan SJA Laboratory Animal Co., Ltd. (Changsha, Hunan, China). The animals were housed under standard conditions (light cycle: 07:00–19:00, temperature: 24 ± 2 °C, humidity: 55 ± 10%) with ad libitum access to food and water. Prior to experimental procedures, the mice were individually handled gently by the experimenter for 5 min per day for one week to reduce handling stress. All animal experiments were conducted in strict accordance with the Guidance for the Care and Use of Laboratory Animals of Shaoyang University and were approved by the Institutional Animal Care and Use Committee (approval date 20 September 2024 and approval code 2024KJKT024).

### 2.2. Drugs

DHM was purchased from Shanghai Yuanye Bio-Technology Co., Ltd (Shanghai, China). and dissolved in 0.9% saline containing 0.1% dimethyl sulfoxide (DMSO). DHM was administered via intraperitoneal injection at a dose of 20 mg/kg once daily at 9:00 AM for 8 consecutive days. The dose of 20 mg/kg (i.p.) was selected based on multiple previous publications demonstrating its efficacy in depression-related behavioral paradigms [10,12,13].

### 2.3. Experimental Design

The animals were randomly divided into three groups: control, CRS, and CRS + DHM (10 mice per group). From day 1 to day 21, mice in the CRS and CRS + DHM groups underwent daily restraint stress, while control mice remained undisturbed in their home cages. From day 15 to day 22, the mice in the CRS + DHM group received intraperitoneal injections of DHM for 8 consecutive days, while mice in the control and CRS groups received intraperitoneal injections of equivalent volume of vehicles (9% saline containing 0.1% DMSO).

**Figure 1:**
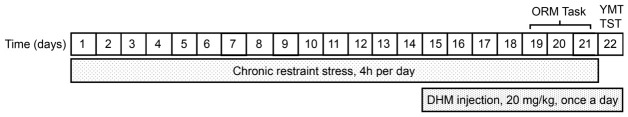
Schematic of animal experimental design displaying the timeline of drug injection and behavior tests. CRS, chronic restraint stress; DHM, dihydromyricetin; ORM, object recognition memory; TST, tail suspension test; YMT, Y-maze test.

The experimental procedure, including the CRS protocol (4 h/day for 21 days), DHM injections (8 consecutive days), and the schedule of behavioral tests, is shown in Figure 1. On days 19, 20, and 21, the object recognition memory (ORM) test was conducted. On day 22, following DHM injection, the Y-maze test and tail suspension test were performed between 12:30 PM and 5:30 PM. The tail suspension test was conducted 2 h after the Y-maze test.

### 2.4. Chronic Restraint Stress

The CRS procedure was based on a previously published protocol [20]. Briefly, mice were individually restrained in plastic tubes (2.7 cm in diameter, 8.15 cm in length) for 4 h per day for 21 consecutive days. After each restraint session, mice were returned to their home cages. The control animals were left undisturbed in their home cages throughout the experimental period.

### 2.5. Object Recognition Memory (ORM) Task

The ORM task was conducted from day 19 to day 21, following procedures described previously [21]. Briefly, each mouse was individually placed into a wooden chamber (30 × 30 × 60 cm) for a 10 min habituation period. Twenty-four hours later, the mice were reintroduced into the chamber containing two identical objects for a 10 min sampling phase. Another 24 h later, the mice were placed in the chamber with one familiar object and one novel object for a 5 min testing phase. The total distance traveled during habituation, sampling, and testing phases was recorded using behavior-tracking software (Any-Maze 6.16; Stoelting Co., Wood Dale, IL, USA). The time spent exploring the familiar object (F) and the novel object (N) during the test phase was analyzed by an experienced experimenter in a double-blind manner. The discrimination index (DI) was calculated as follows: DI = (N − F)/(N + F).

### 2.6. Y-Maze Test

The Y-maze test was performed on day 22, 1 h after DHM injection, as described previously [22]. The mice were placed at the intersection of three arms (40 cm × 8 cm × 20 cm) and allowed to explore freely for 5 min. The total number of arm entries and the spontaneous alternation ratio were recorded and analyzed by an experienced experimenter in a double-blind manner.

### 2.7. Tail Suspension Test

The tail suspension test was conducted on day 22, approximately 2 h after the Y-maze test, according to previously established methods [22]. Briefly, the mice were individually suspended by the tail (approximately 1 cm from the tail tip) from a bar positioned approximately 25 cm above the surface. Each mouse was tested for 6 min, and the immobility time during the final 4 min was recorded and analyzed by an experienced experimenter in a double-blind manner.

### 2.8. Network Pharmacology Analysis

The canonical SMILES string of DHM was obtained from the PubChem database (https://pubchem.ncbi.nlm.nih.gov/, accessed on 5 March 2025). Potential DHM targets were identified using the Super-PRED database (https://prediction.charite.de/, accessed on 5 March 2025; probability threshold > 60%) and the BATMAN-TCM database (http://bionet.ncpsb.org.cn/batman-tcm/, accessed on 5 March 2025; score threshold > 3). Targets associated with memory impairment and depression were retrieved from the GeneCards database (https://www.genecards.org/, accessed on 5 March 2025; score > 3) and the DisGeNET database (https://digenet.com/, accessed on 5 March 2025; all targets selected) using “memory deficit,” “memory impairment,” and “depression” as search keywords. All potential DHM targets were standardized using the UniProt database (https://www.uniprot.org/, accessed on 5 March 2025).

Intersection targets between DHM and the disease-related targets were identified and visualized using Venny 2.1 (https://bioinfogp.cnb.csic.es/tools/venny/, accessed on 5 March 2025). The overlapping targets between DHM, memory impairment, and depression were used to construct a target disease network, which was visualized using Cytoscape software (version 3.8.0; https://cytoscape.org/, accessed on 6 March 2025).

To identify core targets, the intersection targets were submitted to the STRING database (https://string-db.org/, accessed on 6 March 2025) to construct a PPI (protein–protein interaction) network. The species was restricted to *Homo sapiens*, the minimum required interaction score was set to ≥0.4, and isolated nodes were excluded. The resulting PPI data were exported in Tab-Separated Values (TSV) format and imported into Cytoscape (version 3.8.0) for network construction and visualization.

To elucidate the biological functions and signaling pathways of the potential anti-memory impairment and anti-depression targets of DHM, Gene Ontology (GO) and Kyoto Encyclopedia of Genes and Genomes (KEGG) pathway enrichment analyses were performed using R Studio software (2024.04.2-764) with R language (version 4.4.1) (https://www.r-project.org/, accessed on 5 March 2025) and the clusterProfiler R package (https://bioconductor.org/packages/release/bioc/html/clusterProfiler.html/, accessed on 7 March 2025). A *p* value < 0.05 was considered statistical significance. The results were visualized using R studio software (2024.04.2-764).

### 2.9. Molecular Docking Analysis

The molecular docking analysis was carried out using AutoDock Vina (version 1.2.0) [23]. The molecular structure of DHM was retrieved from the PubChem Compound database [24]. Based on degree centrality scores from the PPI network, key targets were selected for molecular docking. The 3D structures of NOS1 (PDB ID: 6CID; resolution: 1.75 Å), ESR1 (PDB ID: 7BAA; resolution: 1.10 Å), CREB1 (PDB ID: 5ZKO; resolution: 3.05 Å), CFTR (PDB ID: 7QI1; resolution: 1.76 Å), GRIA2 (PDB ID: 5ZG2; resolution: 1.25 Å), and TLR4 (PDB ID: 2Z62; resolution: 1.70 Å) were obtained from the RCSB Protein Data Bank (http://www.rcsb.org/, accessed on 10 March 2025). Prior to molecular docking, the structure of related proteins was processed by removing water molecules and adding polar hydrogen atoms. Both protein and ligand structures were converted to PDBQT format. The grid box was centered to encompass the active binding pocket of each protein. AutoDock Tools (version 1.5.6) was used to perform docking calculations and evaluate binding energies. Molecular interactions and binding modes were visualized using BIOVIA Discovery Studio Visualizer (version 2019) and PyMOL 3.0.3.

### 2.10. Molecular Dynamics Simulation

The receptor–ligand complexes obtained from the molecular docking analysis were subjected to molecular dynamics (MD) simulations. The system was parameterized with the AMBER99SB-ILDN force field. The topology for each protein was generated using GROMACS (version 2020.3) with the AMBER99SB-ILDN force field (amber99sb-ildn.ff). The small molecule ligand was parameterized using the ACPYPE tool based on the generalized AMBER force field (GAFF). All systems were solvated in a cubic box with periodic boundary conditions and neutralized by adding appropriate counter ions.

Before production runs, all systems underwent energy minimization with the steepest descent algorithm (5000 steps) to eliminate steric clashes. Subsequently, the systems were equilibrated under conditions of constant volume, temperature and number of particles at 300 K for 100 ps with position restraints applied to the protein–ligand complex, followed by equilibration of constant pressure, temperature and number of particles at 1 bar and 300 K for another 100 ps. Following equilibration, unrestrained MD simulations were performed for 100 ns. Post-simulation analyses included calculations of root mean square deviation (RMSD), root mean square fluctuation (RMSF), radius of gyration (Rg), hydrogen bond analysis, and Gibbs free energy landscape (FEL) reconstruction using GROMACS built-in analysis tools and in-house scripts.

### 2.11. Western Blotting

Hippocampal tissues were isolated and homogenized in RIPA (radioimmunoprecipitation assay) lysis buffer (Thermo Fisher Scientific, Waltham, MA, USA) containing proteinase inhibitors. Protein concentration of individual samples was measured using a bicinchoninic acid protein assay kit (Thermo Fisher Scientific, UK). A total of 30 μg proteins of each sample were separated through SDS-PAGE, then transferred onto PVDF membranes. The membranes were blocked with 5% skimmed milk powder in TBST and incubated overnight at 4 °C with the following primary antibodies: BDNF (Abcam, Cambridge, UK; 1:1000); PKAα (Proteintech, Rosemont, IL, USA; 1:1000); p-CREB (Abcam, Cambridge, UK; 1:5000); CREB (CST, Danvers, MA, USA; 1:1000); and β-actin (Proteintech Europe, Manchester, UK; 1:5000). After washing, the membranes were incubated with the corresponding horseradish peroxidase-conjugated secondary antibodies for 1 h at room temperature. Targeted proteins were visualized using enhanced chemiluminescence reagent and quantified using Quantity One software (version 4.6.2).

### 2.12. Statistical Analysis

All data went through a Shapiro–Wilk normality test and were not significantly deviated from normal distribution (*p* > 0.05). The data were displayed as mean ± standard error of the mean (SEM) and were analyzed using SigmaPlot 14.0. One-way analysis of variance (ANOVA) or two-way ANOVA was used to assess differences among the three groups, followed by appropriate post hoc test for multiple comparisons. A *p* value < 0.05 was considered statistically significant.

## 3. Results

### 3.1. DHM Treatment Alleviated CRS-Induced Depression-Like Behavior in Mice

**Figure 2:**
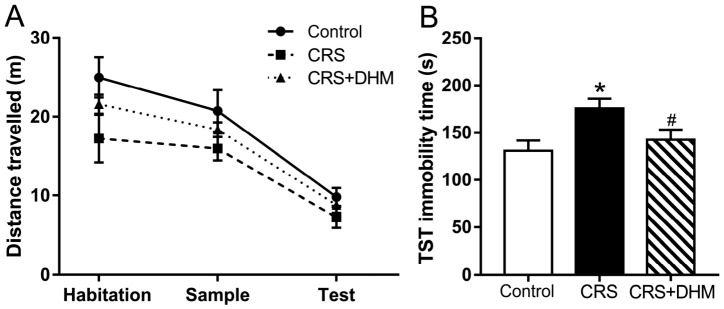
Effect of DHM treatment on depression-like behavior induced by CRS in the tail suspension test (TST). (**A**) The total distance traveled during the habitation, sample, and test phases of the ORM task, reflecting the exploration activity and locomotor function of mice. The data of total traveled distance of each group in habitation phase, sampling phase or testing phase were analyzed through two-way ANOVA, followed by Bonferroni’s post hoc test. There was no significant difference between individual groups in habitation phase, sampling phase or testing phase. (**B**) Immobility time in the TST. Data were expressed as the mean ± SEM (*n* = 10 mice per group). * *p* < 0.05, CRS vs. the control group; # *p* < 0.05, CRS + DHM vs. CRS group. CRS, chronic restraint stress; DHM, dihydromyricetin.

To evaluate the effect of DHM on depression-like behavior and potential locomotor impairment, we performed the tail suspension test. Although the total traveled distance was slightly reduced in the CRS and CRS + DHM groups, there were no significant differences in the total distance traveled during the habitation, sampling, and testing phases among the three groups (Figure 2A). This suggests that neither CRS nor DHM treatment significantly altered the spontaneous exploratory activity or motor functions of the mice, ensuring that the subsequent behavioral changes were not confounded by locomotor deficits.

In the tail suspension test, the CRS group exhibited significantly increased immobility time compared to the control group (*p* < 0.05, Figure 2B), indicating the successful induction of depression-like behavior characterized by increased despair. However, administration of DHM significantly reversed this effect, as evidenced by a marked reduction in immobility time compared to the CRS group (*p* < 0.05, Figure 2B). These results demonstrated that DHM possesses potent antidepressant-like properties in the CRS-induced mouse model.

### 3.2. DHM Alleviates CRS-Induced Recognition and Spatial Memory Impairments

**Figure 3:**
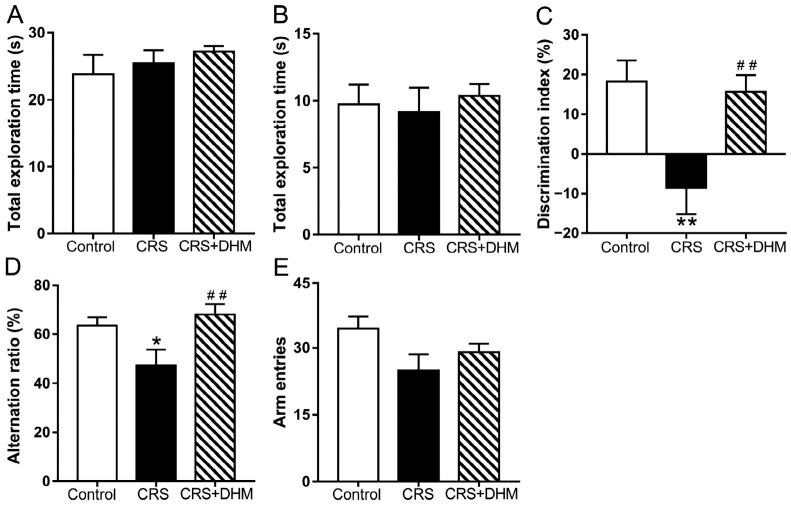
Effect of DHM treatment on CRS-induced memory impairments in ORM and Y-maze tests. (**A**) Total exploration time allocated to identical objects during the sampling (acquisition) phase of the ORM task. (**B**) Total exploration time allocated to both the familiar object and the novel object during the testing phase. (**C**) Discrimination index of three groups. (**D**) Percentage of spontaneous alternation behavior in the Y-maze test. (**E**) Total number of arm entries recorded during the Y-maze test to assess locomotor activity. Data were expressed as the mean ± SEM (*n* = 10 mice per group). * *p* < 0.05 and ** *p* < 0.01, CRS vs. the control group; ## *p* < 0.01, CRS + DHM vs. CRS group. CRS, chronic restraint stress; DHM, dihydromyricetin.

To evaluate the neuroprotective effects of DHM on CRS-induced memory impairment, ORM and Y-maze tasks were conducted. In the ORM task, while total exploration times remained similar across all groups during both the sampling and testing phases (Figure 3A,B), CRS exposure significantly reduced the discrimination index compared with the vehicle group (Figure 3C), indicating a deficit in recognition memory. Notably, DHM administration effectively restored the discrimination index (Figure 3C). Consistently, in the Y-maze test, DHM significantly reversed the CRS-induced reduction in the spontaneous alternation ratio (Figure 3D), while the total number of arm entries remained unchanged among groups (Figure 3E), ruling out confounding effects of locomotor activity. These findings suggest that DHM treatment successfully alleviates CRS-induced spatial and recognition memory impairments.

### 3.3. Common Targets Between DHM and Depression-Related Memory Impairments

**Figure 4:**
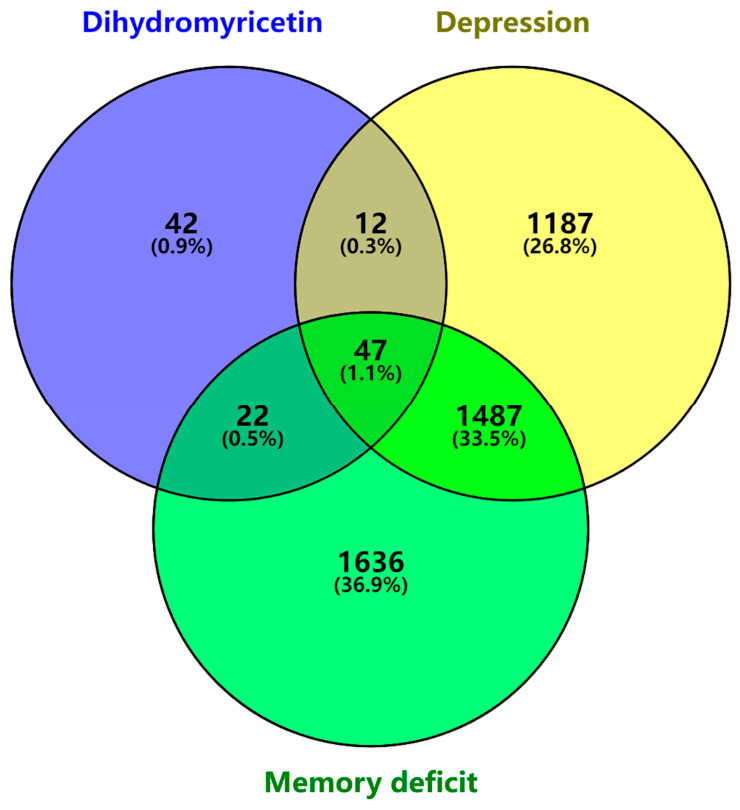
Venn diagram of screened intersection targets of DHM and depression-related memory impairment-related targets.

Using the Super-PRED and BATMAN-TCM databases, we identified 123 potential DHM targets after deduplication. From the GeneCards and DisGeNET databases, we retrieved 3192 memory impairment-related targets and 2733 depression-related targets after deduplication. Venny analysis identified 47 common genes shared among DHM targets, memory impairment targets, and depression targets (Figure 4).

### 3.4. Construction of PPI Network

**Figure 5:**
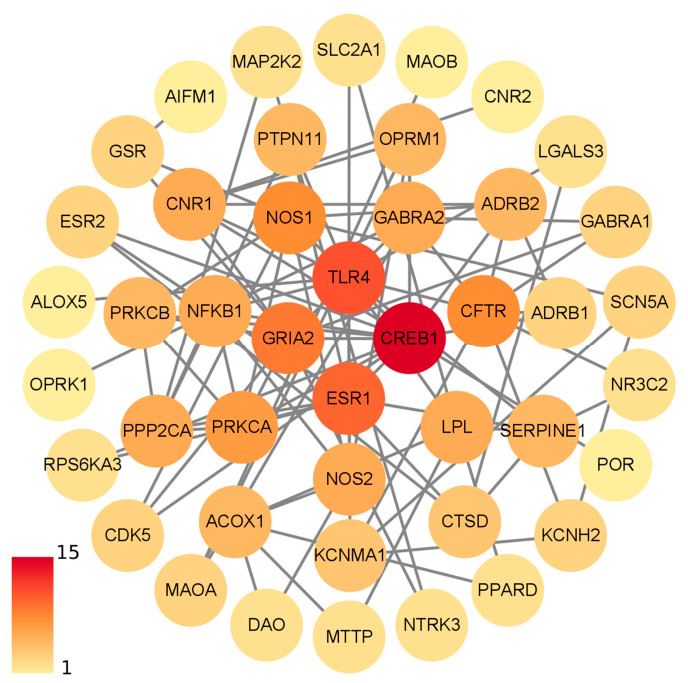
PPI (protein–protein interaction) network of common targets between DHM and depression-related memory impairments.

The 47 intersection targets were imported into the STRING database with interaction confidence > 0.4 for constructing the PPI network, which was visualized using Cytoscape (Figure 5). Node color intensity reflected the number of interactions, with darker colors representing higher connectivity. The resulting network consisted of 47 nodes and 129 edges, with an average node degree of 5.61. The top six nodes ranked by degree centrality were CREB1, TLR4, ESR1, GRIA2, NOS1, and CFTR, suggesting their potential importance in the intersection between DHM’s pharmacological action and the pathophysiology of depression-related memory impairments.

### 3.5. GO and KEGG Enrichment Analyses

**Figure 6:**
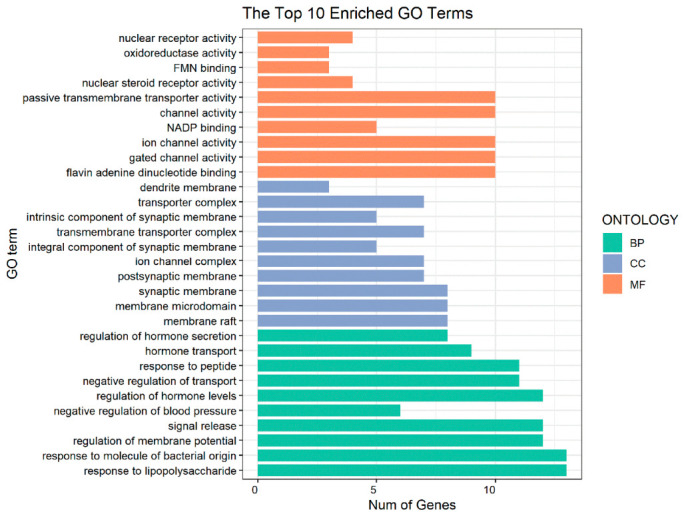
Gene Ontology terms of 47 intersection targets. The top 10 GO functional terms of three categories were shown (adjusted *p* < 0.01). BP: biological processes; CC: cellular component; MF: molecular function.

GO enrichment analysis of the intersection targets yielded a total of 564 biological process (BP) terms, including responses to lipopolysaccharide and bacterial molecules, regulation of membrane potential and hormone homeostasis. A total of 64 cellular component (CC) terms were identified, including synaptic membrane, membrane microdomain, membrane raft and transporter complex. In the molecular function (MF) category, 71 terms were enriched, including activities of channels, transmembrane transporters, receptors and enzymes. The top 10 enriched terms of three GO categories were selected for visualization (Figure 6).

**Figure 7:**
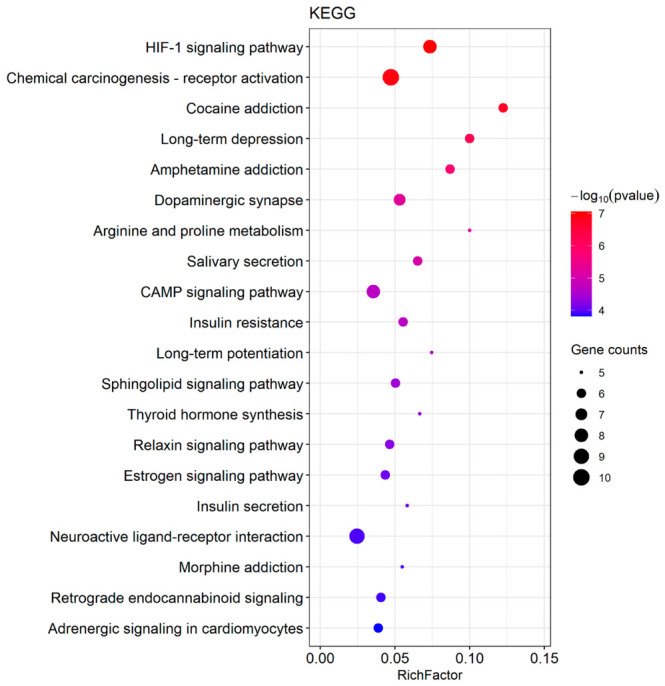
KEGG enrichment of potential targets of DHM and depression-related memory impairments. The diagram showed the top 20 pathways with the highest count values (adjusted *p* < 0.01). Colors and sizes of the spot represent the *p* value and the count value, respectively.

KEGG pathway enrichment analysis further identified multiple signaling pathways potentially involved in the therapeutic action of DHM against depression-related memory impairments (adjusted *p* < 0.05; Figure 7). The enriched pathways included the HIF-1 signaling pathway, cAMP signaling pathway, estrogen signaling pathway, dopaminergic synapse, and neuroactive ligand–receptor interaction, among others. The cAMP signaling pathway, which encompasses the PKA-CREB cascade, emerged as a key pathway of interest. The CREB protein serves as a critical transcription factor downstream of PKA activation, which subsequently regulates the expression of BDNF, a key mediator of synaptic plasticity and long-term memory formation. Based on these findings, we hypothesized that DHM may exert its beneficial effects by targeting key nodes within this pathway, potentially through the PKA-CREB cascade, directly modulating genes associated with synaptic plasticity and memory.

In summary, the network pharmacology analysis identified 47 common targets among DHM, depression and memory deficit, constructed DHM–disease intersection target network, and predicted potential functional pathways.

### 3.6. Prediction of DHM Binding Key Targets

To validate the predicted interactions between DHM and key targets, molecular docking was performed with DHM as the ligand and the six top-ranked proteins from the PPI network as receptors: NOS1 (PDB ID: 6CID), ESR1 (PDB ID: 7BAA), CREB1 (PDB ID: 5ZKO), CFTR (PDB ID: 7QI1), GRIA2 (PDB ID: 5ZG2), and TLR4 (PDB ID: 2Z62).

Binding energy serves as an indicator of ligand–receptor affinity, with lower values representing stronger binding. Generally, a binding energy below −4.25 kcal/mol indicates some binding affinity, below −5.0 kcal/mol suggests good binding capability, and below −7.0 kcal/mol represents strong binding capacity [25]. DHM formed hydrogen bonds with all six key protein targets. Among these, DHM exhibited the strongest binding affinity with CREB1 (−9.225 kcal/mol). Additionally, DHM demonstrated strong binding capacity with NOS1 (−9.113 kcal/mol) (Figure S1).

**Figure 8:**
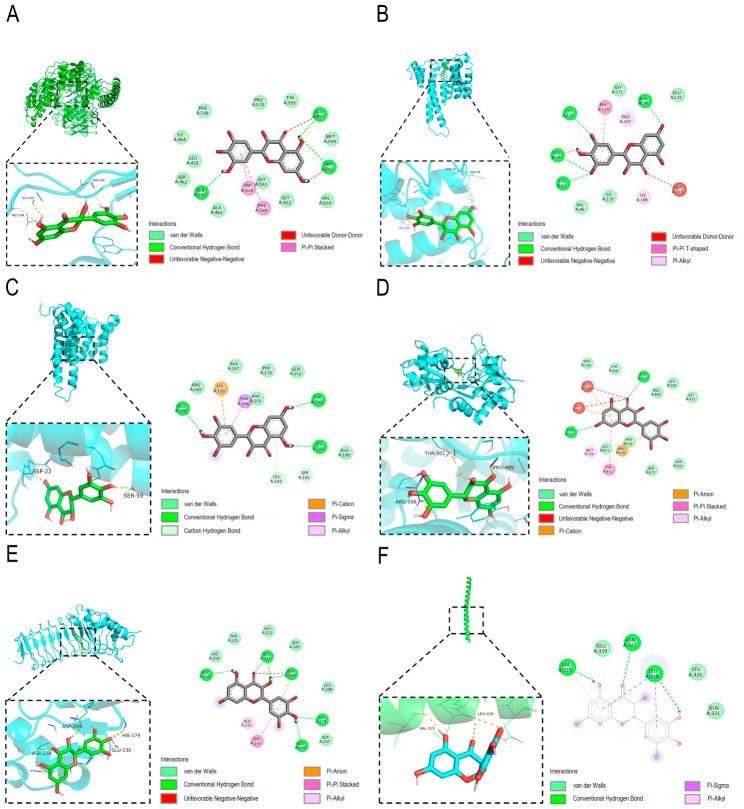
Molecular docking result of DHM and its core targets. (**A**) DHM-NOS1. (**B**) DHM-ESR1. (**C**) DHM-CFTR. (**D**) DHM-GRIA2. (**E**) DHM-TLR4. (**F**) DHM-CREB1.

As illustrated in Figure 8, DHM was bound to key residues of all six protein targets through specific intermolecular interactions. With NOS1, DHM formed aromatic ring stacking interactions with Trp414 and Phe589, conventional hydrogen bonds with Ser590, Trp592, and Glu597, and electrostatic repulsions with Trp592 and Glu597. In ESR1, DHM formed hydrophobic interactions with Ile168 and Pro167; conventional hydrogen bonds with Asn38, Cys42, Ser45, and Lys122; and electrostatic repulsions with Ser45 and Asp215. In CFTR, DHM engaged in hydrophobic interactions with Leu193; conventional hydrogen bonds with Tyr180, Lys189, and Glu200; carbon–hydrogen bonds with Ser192 and Leu193; and electrostatic interactions with Lys159 and Thr196. In GRIA2, DHM formed hydrophobic interactions with Met729; conventional hydrogen bonds with Thr707 and Tyr753; electrostatic interactions with Glu423, Arg506, and Glu726; aromatic ring stacking with Tyr471; and electrostatic repulsions with Glu423 and Glu726. In TLR4, DHM formed hydrophobic interactions with Ala232; conventional hydrogen bonds with His179, Asp209, Ser211, Glu230, and Asp234; electrostatic interactions with Asp209; aromatic ring stacking with Trp256; and electrostatic repulsion with Asp209. Finally, with CREB1, DHM formed hydrophobic interactions with Val315; conventional hydrogen bonds with Val315, Leu318, and Asn322; and electrostatic interactions with Leu318.

These molecular docking results demonstrated that DHM exhibits favorable binding capabilities with the key protein targets identified by network pharmacology, supporting the validity of the network pharmacology-based screening approach.

### 3.7. Molecular Dynamics Simulation Analysis

**Figure 9:**
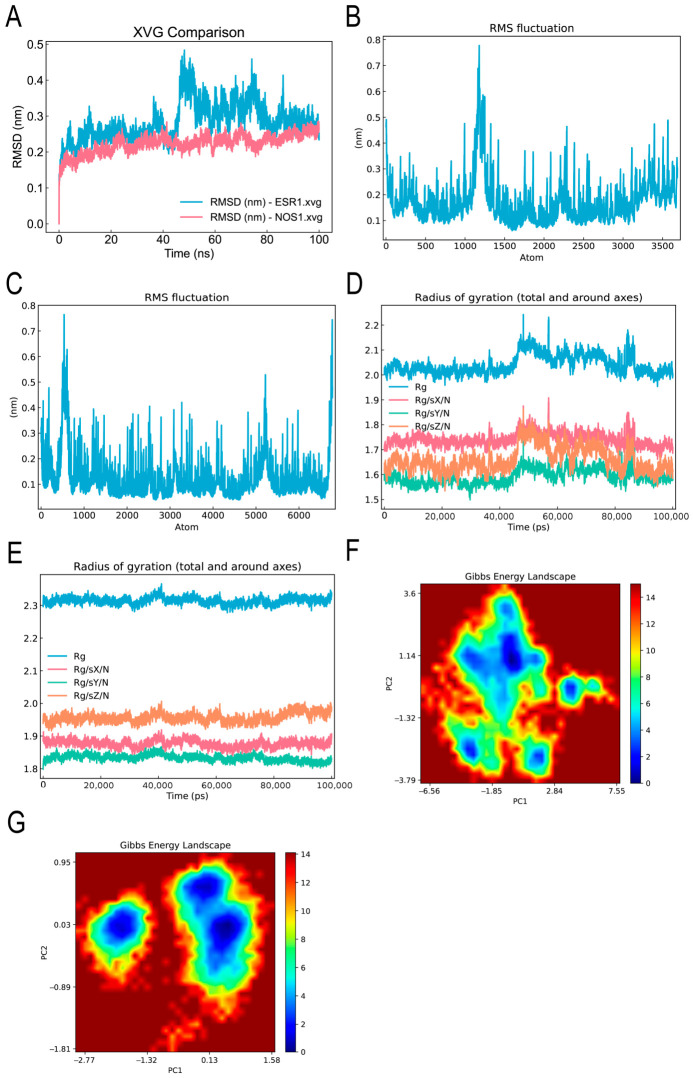
Molecular dynamics simulation results. (**A**) RMSD of DHM-ESR1 complexes and DHM-NOS1 complexes during molecular dynamics simulations. (**B**) RMSF of DHM-ESR1 complexes during molecular dynamics simulations. (**C**) RMSF of DHM-NOS1 complexes during molecular dynamics simulations. (**D**) Radius of gyration of DHM-ESR1 complexes during molecular dynamics simulations. (**E**) Radius of gyration of DHM-NOS1 complexes during molecular dynamics simulations. (**F**) Energy landscape of DHM-ESR1 complexes during molecular dynamics simulations. (**G**) Energy landscape of DHM-NOS1 complexes during molecular dynamics simulations.

Molecular dynamics simulations confirmed the stability of DHM binding to the core targets (Figure 9A). RMSD measures the structural deviation of a conformation relative to the initial structure over time; lower RMSD values indicate more stable binding conformations. The results demonstrated stable binding of DHM to each core target, with RMSD fluctuations converging to a range of 0.4–0.5 nm after approximately 50 ns of simulation (Figure 9B,C), indicating stable and sustained ligand–target interactions.

The radius of gyration (Rg) measures the compactness and structural folding stability of the protein–ligand complex during simulation. As shown in Figure 9D,E, the Rg values for DHM complexed with each target remained within 2.5 nm throughout the 100 ns simulation, indicating that all complexes maintained structural compactness.

The free energy landscape (FEL), constructed based on the principal components PC1 and PC2, was used to characterize the energetically favorable conformational states of the protein–ligand complexes. Figure 9F,G show that the DHM-ESR1 and DHM-NOS1 complexes each exhibited multiple minimum energy basins. This finding indicates that upon DHM binding, both ESR1 and NOS1 adopt multiple stable conformational states, demonstrating the conformational diversity and flexibility of the ligand–protein system.

Taken together, the MD simulation results demonstrated that DHM binds stably to the identified core targets, supporting the potential of DHM as a multi-target therapeutic agent for depression-related memory impairments.

### 3.8. DHM Treatment Alleviated CRS-Induced Downregulation of the PKA-CREB-BDNF Pathway in Mouse Hippocampus

**Figure 10:**
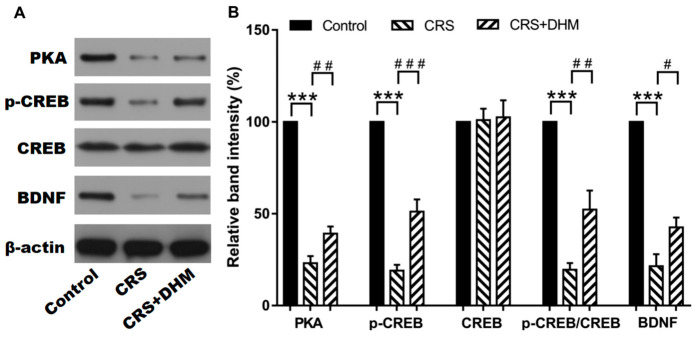
Effect of DHM treatments on expression of PKA-CREB-BDNF signaling pathway components in CRS mouse hippocampus. (**A**) Representative Western blot of hippocampal PKAα, p-CREB, CREB, BDNF in three animal groups from Figure S2-5. (**B**) Quantification for band intensities of PKAα, p-CREB, CREB, BDNF, normalized to β-actin; p-CREB was also normalized to CREB. Data was presented as mean ± SEM (*n* = 5 animals per group). *** *p* < 0.001, vs. control group, ### *p* < 0.001, ## *p* < 0.01, # *p* < 0.05, vs. CRS group. CRS, chronic restraint stress; DHM, dihydromyricetin.

To validate the network pharmacology prediction that the PKA-CREB-BDNF pathway mediates the therapeutic effects of DHM on depression-related memory impairment, we examined the hippocampal expression levels of PKAα, p-CREB, CREB, and BDNF with Western blot (Figure 10 and Figure S2-5). CRS exposure alone resulted in significant decreases in hippocampal PKAα, p-CREB, and BDNF protein expression levels compared to that of the control mice (*p* < 0.05); DHM co-treatment significantly upregulated the expression of those proteins (*p* < 0.05). Additionally, neither CRS exposure nor DHM co-treatment significantly affected the level of total CREB protein (*p* = 0.167), indicating that the observed effect was specific to CREB phosphorylation rather than total CREB protein levels. These results suggest that DHM amelioration of CRS-induced behavioral deficits is associated with restoration of the hippocampal PKA-CREB-BDNF signaling pathway.

## 4. Discussion

In the present study, we investigated the neuroprotective effects of DHM on CRS-induced comorbid depression and memory impairments. DHM treatment significantly alleviated depression-like behaviors and restored both recognition and spatial working memory without altering spontaneous locomotor activity. These findings underscore the therapeutic potential of DHM in addressing depression-associated cognitive deficits—an area where conventional antidepressants often show limited efficacy.

The tail suspension test is a widely used paradigm for evaluating behavioral despair and screening antidepressant compounds [26,27]. CRS-exposed mice exhibited significantly prolonged immobility time, consistent with previous reports that CRS induces depression-like behaviors in rodents [19]. DHM treatment effectively reduced immobility, demonstrating clear antidepressant-like effects. However, more depression-like behavioral tests, such as sucrose preference, forced swimming and social interaction tests, will further accredit DHM’s capacity against depression. Total distance traveled in the ORM task and total arm entries in the Y-maze task confirmed that neither CRS nor DHM affected locomotor activity, validating the specificity of DHM’s antidepressant-like properties. These results align with previous studies, showing that DHM ameliorated hippocampal astrocyte damage in diabetic neuropathic pain–depression comorbidity models [9] and mitigated LPS-induced depressive-like behavior [11].

Memory impairment is a clinically significant symptom of depressive disorder. Consistent with previous reports that various stress paradigms induce memory deficits [3], we found that CRS impaired both long-term non-spatial memory (ORM task) and short-term spatial working memory (Y-maze task). The DHM treatment effectively rescued both impairments. Importantly, locomotor activity and exploratory behavior remained unchanged across groups, confirming that DHM ameliorates memory deficits through cognitive mechanisms rather than nonspecific motor or motivational effects.

To elucidate the underlying mechanisms, we applied an integrated strategy combining network pharmacology, molecular docking analysis, and molecular dynamics simulations. Network pharmacology identified 47 intersecting targets between DHM and depression-related memory impairment, with PPI topological analysis highlighting CREB1, ESR1, and NOS1 as core hub nodes. KEGG enrichment suggested that the action of DHM against depression-associated memory impairment is possibly linked to multiple signaling pathways, some of which modulate synaptic plasticity and memory cascades. Molecular docking revealed strong binding affinities between DHM and its core targets, particularly CREB1 and NOS1. Subsequent 100 ns molecular dynamics simulations thermodynamically validated DHM as a stable multi-target ligand, providing a solid theoretical foundation for its *in vivo* efficacy.

The cAMP-PKA-CREB-BDNF signaling pathway is a critical mediator of neuroplasticity implicated in both depression and memory regulation. CREB, a key transcription factor, links neuronal activity to downstream gene expressions including BDNF, which supports synaptic plasticity, long-term potentiation, and memory consolidation. Chronic stress suppresses CREB phosphorylation and reduces hippocampal BDNF expression, contributing to synaptic atrophy and cognitive decline [28,29]. ESR1 serves as a critical upstream modulator capable of activating CREB signaling, and its downregulation is closely associated with CRS-induced behavioral despair and memory deficits [30,31]. In the central nervous system, CREB and NOS1 form a critical bidirectional feedback loop that regulates neuroplasticity, neurogenesis, learning and memory [32]. The structural stability of DHM binding to ESR1, NOS1, and CREB1, as confirmed by our docking and the dynamics simulations for ESR1 and NOS1, provides a mechanistic basis for DHM’s ability to simultaneously modulate estrogenic signaling and CREB-mediated neurotrophic gene expression, thereby exerting dual antidepressant and cognitive-enhancing effects. Consistent with these computational predictions, our in vivo experiments confirmed that DHM significantly upregulated hippocampal PKA, p-CREB, and BDNF expression in CRS-exposed mice. The Western blot analysis demonstrated that DHM effectively restores PKA-mediated CREB phosphorylation and subsequent BDNF expression, rescuing the stress-induced impairment of the hippocampal signaling axis. These results suggest that DHM ameliorates CRS-induced behavioral deficits possibly through targeting and reactivating the PKA-CREB-BDNF pathway, consistent with an early report showing DHM alleviated sleep-deprivation-induced memory impairment via activating the same pathway [17]. Furthermore, the PKA-CREB-BDNF signaling cascade has been implicated in various forms of stress-induced memory impairment, including chronic unpredictable mild stress and hypobaric-hypoxia-induced depression models [18,33], further supporting our findings.

Our findings align with accumulating evidence on related compounds. The rapid antidepressant efficacy of Fructus Aurantii, Kamishoyosan, and XingPiJieYu [29,34,35], as well as the neuroprotective effects of p-coumaric acid [36], have all been attributed to activation of the PKA-CREB-BDNF pathway. The present study extends these findings by demonstrating that DHM, with its structurally distinct dihydroflavonoid scaffold, exerts beneficial effects via the same core pathway while acting on multiple targets as predicted by network pharmacology. This suggests that the PKA-CREB-BDNF axis represents a convergence point for multiple neuroprotective compounds targeting comorbid depression and cognitive dysfunction [37,38,39].

Inflammation has been widely recognized as a major contributor to the pathophysiology of depression [40]. TLR4, a core target of DHM, is a depressive disorder gene and high level of TLR4 expression in central nervous system is associated with major depression disorder [41]. TLR4 can activate the NF-κB (a common target of DHM) pathway, releasing proinflammatory cytokines and resulting in impaired neurogenesis in the hippocampus, neurotransmitter disruption and reduced BDNF, which contribute to depression and memory impairment [42,43,44,45]. DHM has been shown to alleviate cognition deficit in Alzheimer’s disease mice [46] and depression-like behavior in LPS-treated mice [11,12], respectively, via inhibiting TLR4-mediated neuroinflammation. Additionally, TLR4-RAGE crosstalk regulates LPS-induced inflammation in macrophages and DHM has shown to suppress the AGE-RAGE pathway and mitigate corticosterone-indued depressive-like behaviors [13]. TLR4 also regulates astrocyte-related signal pathways, which are involved in depression [47]; Watanabe et al. reported that DHM relieved social-isolation-induced memory decline in mice via restoring astrocyte plasticity [16]. Therefore, it is reasonable that alleviation of depression-like behavior and memory impairment in the CRS mice by DHM is at least partially through inhibiting TLR4-mediated neuroinflammation, which requires further investigation.

Several limitations warrant consideration. First, while MD simulations confirm the thermodynamic stability of DHM-target binding, further experiments (e.g., the cellular thermal shift assay) are required to validate target occupancy in living tissues. Second, although the bioinformatical analysis predicted that there are 47 targets of DHM and that DHM directly interacts with the six core targets, we only validated the PKA-CREB-BDNF pathway; the specific contributions of other predicted targets (for example, ESR1, NOS1, TLR4, etc.) warrant investigation using appropriate cell lines and animal models with different approaches, e.g., pharmacological inhibition of targeted pathways. Third, though a single dose of DHM (20 mg/kg) has shown protection against depression-related disorders by our groups and other researchers, future research should explore the effects of dose–response and time–course on depression-like behaviors and biochemical changes in predicted signal pathways. Fourth, we only used male mice in this study due to traditionally estrous cycles in female mice may cause data variability, but recent studies have clearly demonstrated that when assessing depression-like behaviors through tail suspense, forced swim and the sucrose preference tests in rodents, there were no sex differences in C57BL/6N, DBA/2, or FVB/N strains; however, there are sex difference on the baseline apathy-like behaviors depending on the mouse strain [48,49]. Therefore, we plan to also include female mice in future studies to validate the current findings across both sexes and explore potential sex differences. Finally additional mechanisms underlying DHM’s antidepressant effects, including neuroinflammation, oxidative stress, and adult hippocampal neurogenesis, remain to be comprehensively investigated.

## 5. Conclusions

In conclusion, DHM alleviates CRS-induced depression-like behaviors and memory deficits in mice, possibly via multiple signaling pathways, including the confirmed hippocampal PKA-CREB-BDNF signaling pathway. Our integrated computational and biochemical analyses revealed that DHM acts as a stable multi-target ligand for CREB1, ESR1, NOS1 and TLR4, and possibly effectively reverses the suppression of PKA, p-CREB, and BDNF to promote synaptic plasticity, which requires further validation. These results highlight DHM as a promising natural therapeutic candidate for depression-associated cognitive impairment, supporting its further clinical development.

## Data Availability

The raw data contributed to this article will be made available by the corresponding authors upon request.

## Supplementary Materials

The following supporting information can be downloaded at: https://www.mdpi.com/article/10.3390/biology15141144/s1, Figure S1: The heat map of the molecular docking scores of DHM and its targets; Figure S2: Original images of Western blot.

## Abbreviations

The following abbreviations are used in this manuscript:

AGE	advanced glycation end-products
BDNF	brain-derived neurotrophic factor
BP	biological process
CC	cellular component
CFTR	cystic fibrosis transmembrane conductance regulator
CREB	cAMP response element-binding protein
p-CREB	Phosphorylated cAMP response element-binding protein
CRS	chronic restraint stress
DHM	dihydromyricetin
ESR1	estrogen receptor alpha
KEGG	Kyoto Encyclopedia of Genes and Genomes
GO	Gene Ontology
GRIA2	glutamate receptor ionotropic AMPA 2
LPS	lipopolysacchride
MD	molecular dynamics
MF	molecular function
NOS1	nitric oxide synthase 1
ORM	object recognition memory
PKA	protein kinase A
PPI	protein–protein interaction
RAGE	receptor for advanced glycation end-products
