# Supplementary material for: Dihydromyricetin Mitigates Depression-Related Memory Impairments Through Regulation of Hippocampal PKA-CREB-BDNF Pathway in Mice
Source: Biology (Basel). 2026 Jul 14;15(14):1144. doi: 10.3390/biology15141144 (PMC13405890; doi:10.3390/biology15141144)
Supplement: Supplementary file 1 [file biology-15-01144-s001.zip › biology-4402916-supplementary.pdf]

Supplementary data Figure S1

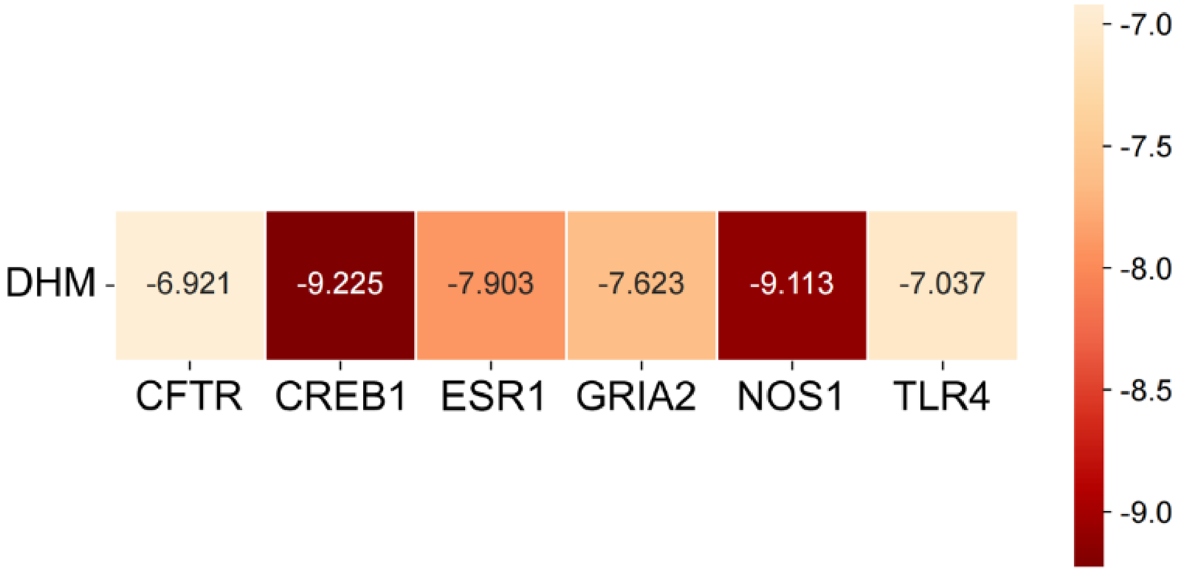

Figure S1 The heat map of the molecular docking scores of DHM and its targets. DHM, Dihydromyricetin.

Supplementary data Figure S2

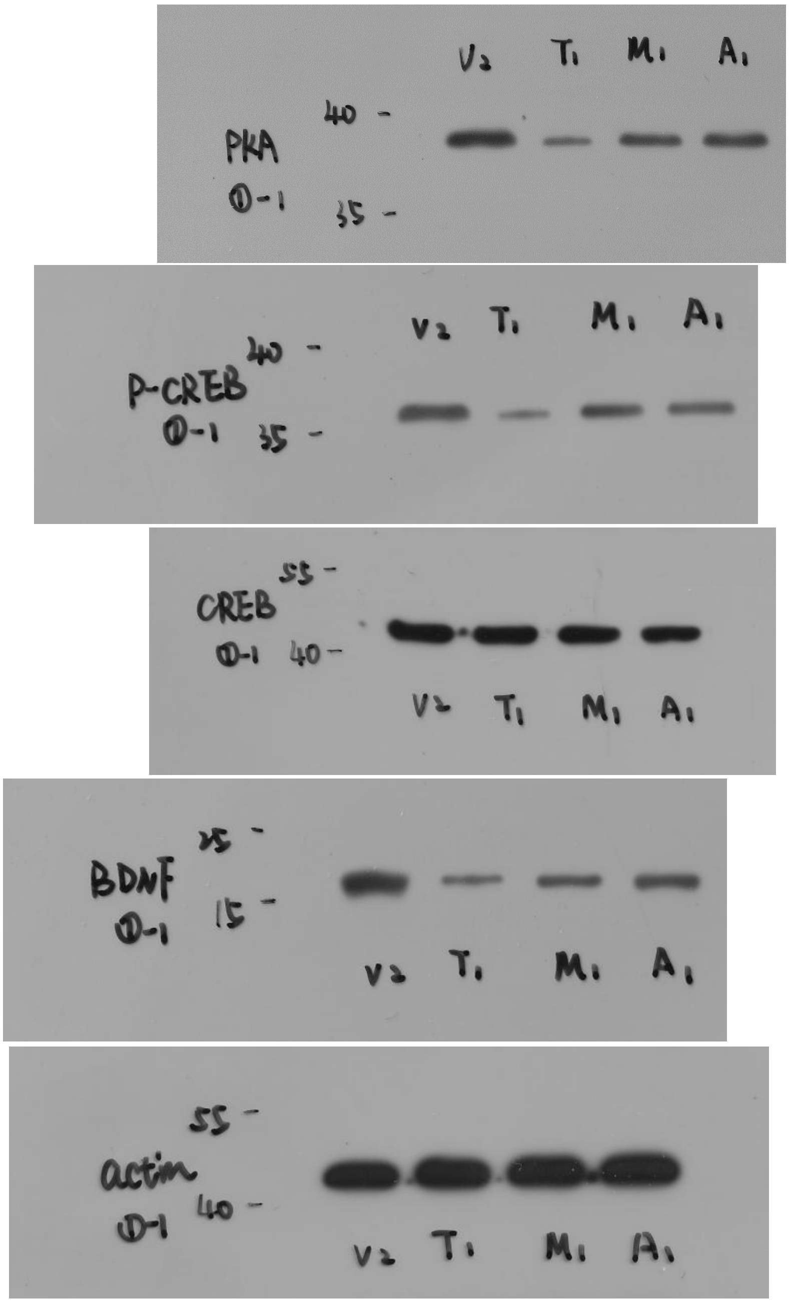

Figure S2-1. Original images of western blot (sample one of each group). V2, control group; T1, chronic restraint stress (CRS) group; M1, CRS+DHM (dihydromyricetin) group; A1, a sample for another project.

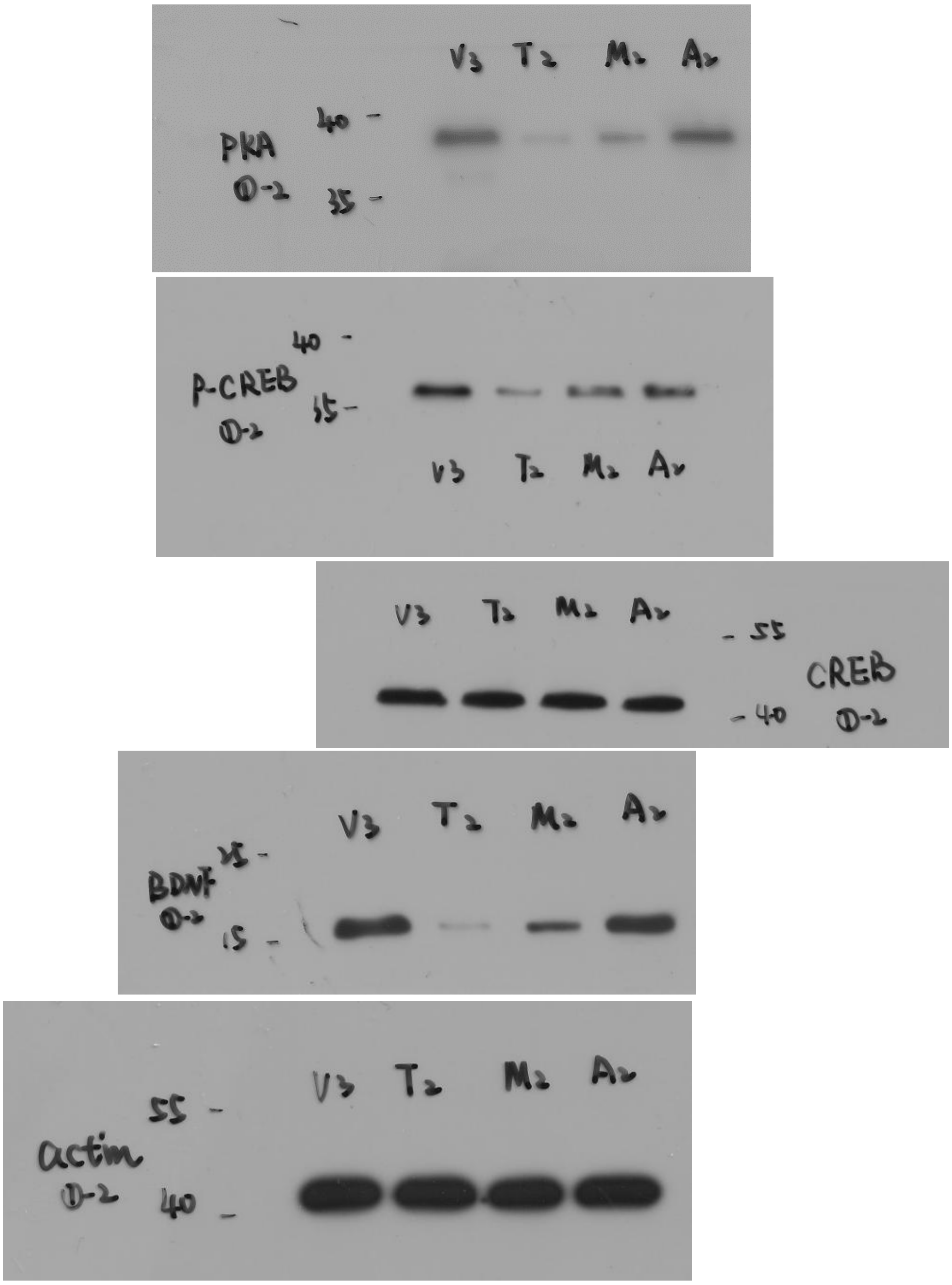

Figure S2-2 Original images of western blot (sample 2 of each group). V3, control group; T2, chronic restraint stress (CRS) group; M5, CRS+DHM (dihydromyricetin) group; A2, a sample for another project.

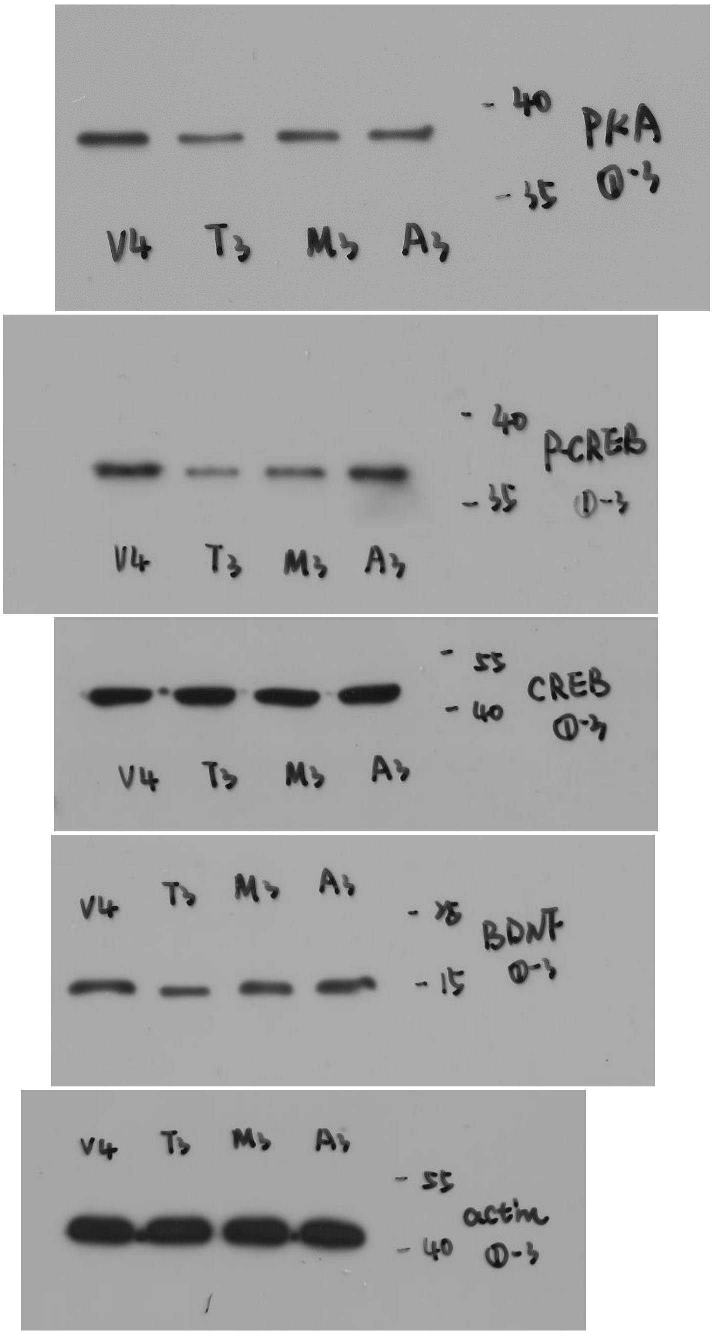

Figure S2-3 Original images of western blot (sample 3 of each group). V4, control group; T3, chronic restraint stress (CRS) group; M5, CRS+DHM (dihydromyricetin) group; A3, a sample for another project.

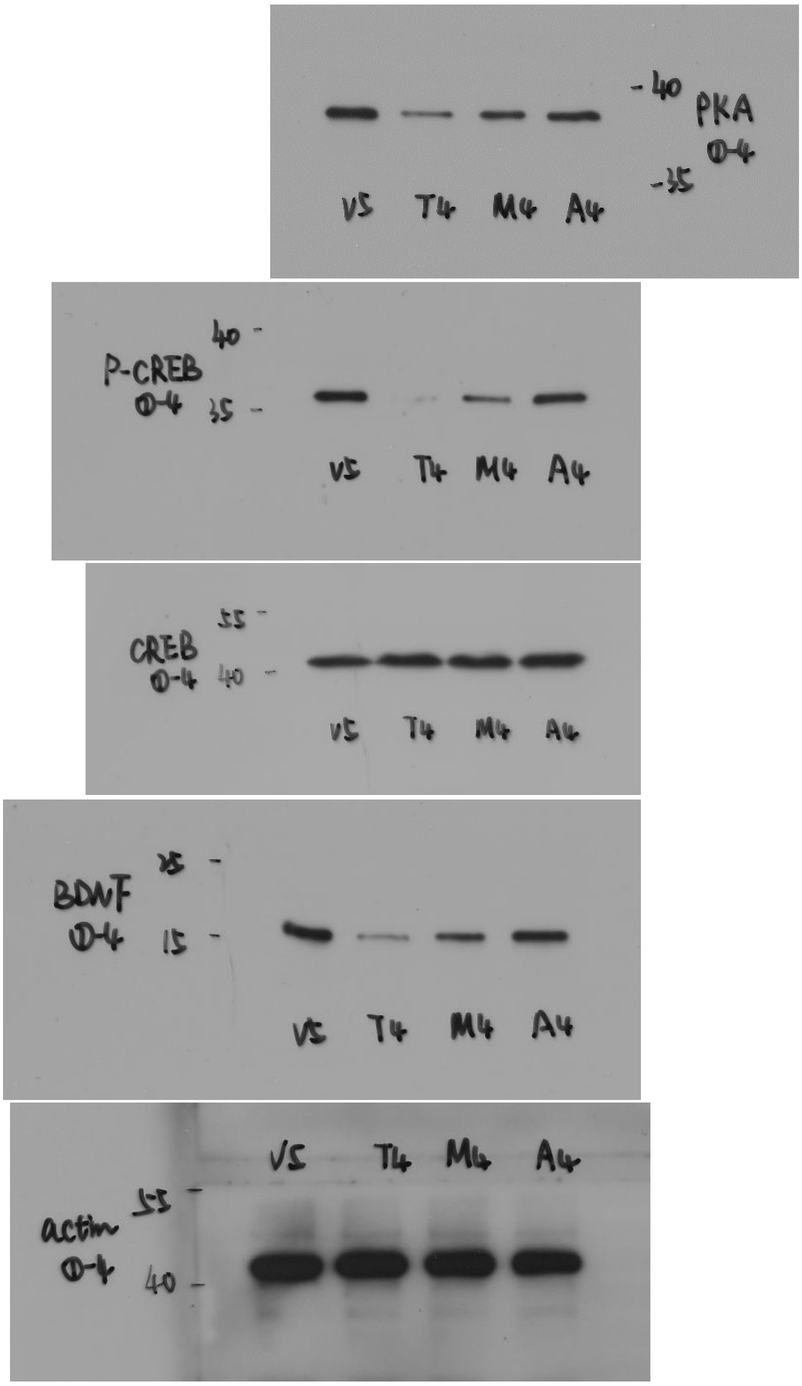

Figure S2-4 Original images of western blot (sample 4 of each group). V5, control group; T4, chronic restraint stress (CRS) group; M4, CRS+DHM (dihydromyricetin) group; A4, a sample for another project.

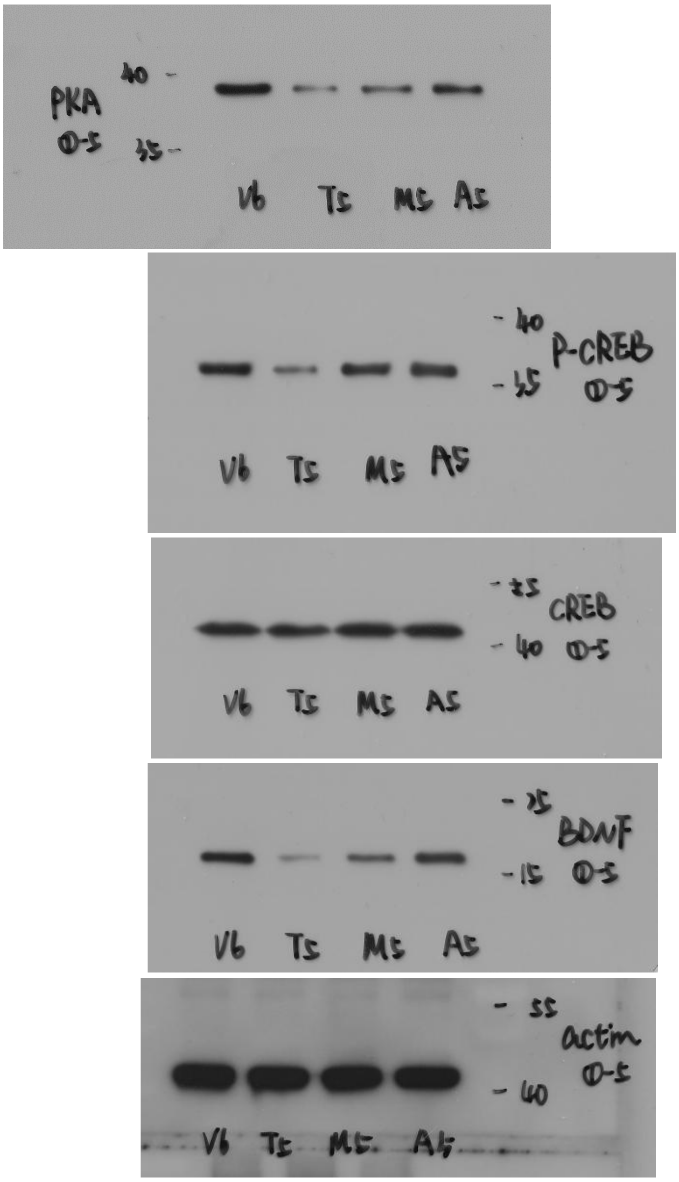

Figure S2-5 Original images of western blot (sample 4 of each group). V6, control group; T5, chronic restraint stress (CRS) group; M5, CRS+DHM (dihydromyricetin) group; A5, a sample for another project.
